# Identification and modelling of dynamic parameters for round link chains subject to axial loads

**DOI:** 10.1038/s41598-022-19207-3

**Published:** 2022-09-28

**Authors:** Kun Zhang, Zhengxian Sun, Jinpeng Su, Xuntao Wei, Mingchao Du, Hongyue Chen

**Affiliations:** 1 Shandong Provincial Key Laboratory of Robotics and Intelligent Technology, Shandong University of Science and Technology, Qianwangang Road 579, Qingdao, 266590 Shandong Province China; 2 Shandong Energy Group, Jingshi Road 10777, Jinan, 250014 Shandong Province China; 3 School of Mechanical Engineering, Liaoning Technical University, Yulong Road 88, Fuxin City, 123000 Liaoning Province China

**Keywords:** Engineering, Mechanical engineering

## Abstract

A round link chain subject to axial dynamic loads composes a nonlinear viscoelastic system. Unlike the classical pounding problems, the round link chain will not only suffer linear elastic deformation, but also nonlinear plastic or impacting deformation. Based on theoretical formulation and experiments, a new approach is presented in this paper to model and identify the nonlinear dynamic parameters, namely the stiffness and damping for the round link chain. With linear deformation, nonlinear deformation and energy dissipation considered, a modified nonlinear viscoelastic model is developed to describe the vibrational behavior of the chain with numbers of round links. The linear elastic model and impacting model are combined to derive the equivalent nonlinear stiffness, while experiments and the least square fitting method are employed to identify the nonlinear damping according to the modified nonlinear viscoelastic model. The influences of the key parameters such as the length of the chain, elastic module and loading frequency on the dynamic stiffness and damping are investigated. Another test is performed to validate the identification model and good agreements are observed.

## Introduction

As key components of shipping/lifting hoists or bulk material handling machines, round link chains are wildly used in marine, mechanical, mining and civil engineering. Investigations on the dynamic characteristics are of great significance for good performance of the corresponding equipment and machines^1–3^.

Numerous researchers have been devoting to the static and dynamic analysis for kinds of round link chains. Ming et al.^4^ derived an equation to relate the contact area of the round link chains to the static contacting stress. Li et al.^5^ calculated the maximum stress and pressure distribution of the contact area of the ring chain by Hertz theory, and obtained the maximum stress of the meshing contact area of the sprocket chain. Bian et al.^6^ established the mathematical equation of circular chain structure, deduced two static analysis models of chain-ring contact, simulated and analyzed the collision process between circular chains, and revealed the fatigue fracture mechanism and fatigue crack propagation law of circular chain. Li et al.^7^ established a virtual prototype model for dynamics simulation of chain drive system, simulated and analyzed the start-up of ring chain load after shutdown, and obtained the variation law of kinematics and dynamic behavior response of meshing contact. Diao et al.^8^ conducted photo-elasticity experiments and finite element (FE) analysis on the contact stress for the round link chains. Wang et al.^9^ used time-varying dynamic analysis method to obtain the dynamic tension distribution of the chain of heavy scraper conveyor. The finite element analysis was carried out on the three-dimensional contact between the adjacent chains of the straight segment and the bending segment to obtain the three-dimensional stress distribution of the chain.

As reviewed, since the round link chain usually suffer heavy axial load to convey bulk material or to transfer motions, reasonably more attentions are paid to the dynamic characteristics in the axial direction. When the round chain is subjected to harmonic or impacting excitations, impacting, friction and even plastic deformation^10^ can be observed. The round link chain composes a typical viscoelastic system. The damping effects and the nonlinear stiffness should be considered in the dynamic analysis. The Kelvin–Voight model, which was firstly developed for the pounding problems^11^, is widely used to take the energy dissipation into account. However, quite similar to the pounding problems, the contact, friction and the plastic deformation are actually all nonlinear. Thus, the linear model cannot totally consider the nonlinear factors for the damping and stiffness. To address the issues for the pounding problems, kinds of non-linear models^12^, such as the Hertzdamp non-linear model^13^ and nonlinear Hunt–Crossley model^14^ have been proposed. All the nonlinear models are quit efficient and accurate for lots of cases and they are also well employed to model other viscoelastic systems^15–17^.

However, the stiffness and damping coefficients can hardly be determined for complex practical systems. Thereby, considerable work has been done to develop identification methods and experimental techniques to identify or measure the dynamic parameters^18^. Based on the servo motor current and corresponding position deflection, Yang et al.^19^ developed a new identification methodology for the accurate joint stiffness of heavy duty robots. Fan et al.^20^ employed the expected trajectory and external load to identify the stiffness defect of a parallel manipulator. Jin et al.^21^ extended the Hertzdamp model to derive the equivalent stiffness and damping for auto eliminating clearance auxiliary bearing devices. Liu et al.^22^ established a variable motion mapping method to enhance stiffness identification of the remote object. Based on the output properties, Zhao et al.^23^ developed an algorithm to reconstruct simultaneously an anti-damping coefficient for some anti-stable systems, yet the algorithm is not suitable for the Kelvin–Voigt damping and some other spatially varying damping.

With the devolvement of experimental techniques, more and more test-based composite methods are proposed to obtain the dynamic stiffness and damping. Wang et al.^24^ designed a measurement system and derived a tangential contact stiffness model to identify the friction damping and the key parameters of tangential contact stiffness in the transition process. Xu et al.^25–27^ developed a laser-based deformation modeling methods and successfully identified complex deformations for composite structures. Lee et al.^28^ utilized curved-edge diffraction to improve the dimensional measurement technique, and the dynamic parameters for a ball bearing spindle system was obtained. Domenico Lisitano et al.^29^ formulated a so called layer method for damping matrix identification using the experimental receptance- -matrix data together with physical connectivity constraints. The method could overcome the ill-conditions and is stable for various frequency ranges. Budak et al.^30^ put forward a new identification framework for the process parameters in turning and milling. The process damping was obtained from chatter tests and the indentation force coefficient is identified with an energy method. Ben Romdhane et al.^31^ performed experiments to determine the loss factors of a non-obstructive particle system with large number of freedoms. When the damping properties were obtained, they employed an equivalent viscous damping to predict the dynamic characteristics of a beam with the non-obstructive particle system.

The above review indicates that the nonlinear viscoelastic models can be successfully used to study the dynamic characteristics of a wide range of viscoelastic systems, not just limited to the pounding problems. Even though the stiffness and damping coefficients are hardly determined theoretically for complex systems, including the round link chain, the model still can provide physical insights into the viscoelastic problems and can be readily employed to develop experiment-based composite identification methods. Moreover, as presented in the latest literature, the stiffness, linear or nonlinear, can be formulated in a theoretical way, though the expressions of the nonlinear ones are quite complex. However, damping properties for complicated systems are usually obtained using tests, because the energy dissipating mechanism for the systems is comprehensive and sophisticated. Hence, this paper is intended to combine the theoretical modeling and experiments for dynamic parameter identification of a round link chain with numbers of round links. An energy method is utilized to deduce the nonlinear stiffness, including the linear elastic one and the one for the indentations. Based on a modified nonlinear viscoelastic model and testing data, the nonlinear damping for the round link chain is given. The identified results is validated with a new experimental case and the effects of the material and geometrical properties are also examined.

## Modeling for the vibrational stiffness and damping

**Figure 1:**
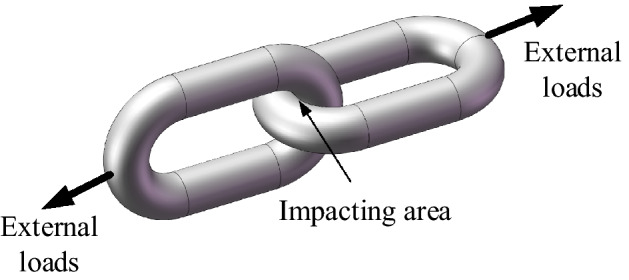
Force diagram of the round link chain.

**Figure 2:**
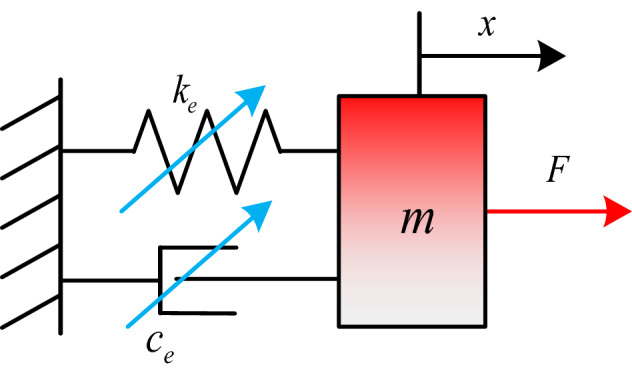
Equivalent dynamic model for a round link chain with *n* links.

As shown in Fig. 1, when a round link chain is subjected to large dynamic loads in the axial direction, impacting, elastic and plastic deformations will occur. It composes a typical viscoelastic system. In order to accurately simulate stiffness effects and energy dissipation, a nonlinear vibrational system is proposed for a round link chain with *n* links in this paper. As shown in Fig. 2, the model is based on the non-linear viscoelastic model in Ref12. In the model, the linear stiffness and nonlinear contact stiffness are modeled as an equivalent nonlinear stiffness. The energy dissipation in the contact process is expressed with equivalent damping. The total mass of the *n* links is simulated with a concentrated mass *m*.

In this way, the governing equations can be expressed as

1 $$F = F_{m} + F_{k} + F_{c}$$

where the inertial force $F_{m} = m\ddot{x}$ and $x$ is the displacement of the chain. The elastic force $F_{k} = k_{e} \left( x \right)x$, $k_{e} \left( x \right)$ is the equivalent nonlinear stiffness. The dissipated force $F_{c} = c_{e} \left( x \right)\dot{x}$ and $c_{e} \left( x \right)$ is the nonlinear equivalent damping. In the model, the total mass *m* is known, while the nonlinear elastic and dissipated forces are needed to be determined.

### Formulation of the equivalent nonlinear elastic force

**Figure 3:**
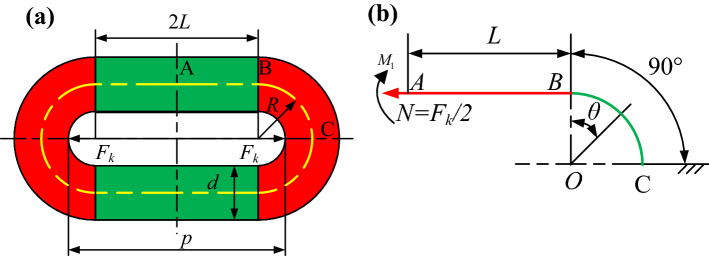
Force diagram of a round link in the chain, (**a**) geometry of the round link and (**b**) simplified force diagram.

As analyzed above, the equivalent nonlinear elastic force $F_{k}$ can be divided into two parts, namely the linear elastic force and the nonlinear elastic force. The linear elastic force is deduced by the linear elastic deformation of the chain, while the nonlinear elastic force is due to the contact between the adjacent round links. One link of the round link chain is taken and analyzed to derive $F_{k}$, as shown in Fig. 3. The round link is assumed to comprise homogeneous and isotropic elastic materials.

For the linear elastic deformation of the round link, the assumption of infinitesimal strain theory is employed. Due to the deformation coupling of the curved segment BC in the round link, the application of axial load $F$ will introduce axial force $N$, bending moment $M$ and shear force $Q$ acting on the straight segment AB. Considering the symmetry of the round link and the axial load, the forces and moment can be defined as

2 $$\left\{ \begin{gathered} M = M_{1} \hfill \\ N = \frac{{F_{k} }}{2} \hfill \\ Q = 0 \hfill \\ \end{gathered} \right.$$

The axial force $N_{c}$, the moment $M_{c}$, and the shear force $Q_{c}$ acting on the curved segments are related to the subtended angle $\theta$ and can be expressed as

3 $$\left\{ \begin{gathered} N_{C} = \frac{{F_{k} }}{2}\cos \theta \hfill \\ M_{C} = M_{1} - \frac{{F_{k} }}{2}R\left( {1 - \cos \theta } \right) \hfill \\ Q_{C} = - \frac{{F_{k} }}{2}\sin \theta \hfill \\ \end{gathered} \right.$$

The total potential energy of the round link is composed of the potential energy $U_{AB}$ stored in the straight segment and the potential energy $U_{BC}$ for the curved segment. Thus, the total potential energy of the round link chain $U$ can be obtained as

4 $$\begin{gathered} U = U_{AB} + U_{BC} = \int_{0}^{L} {\left( {\frac{{N^{2} }}{{2EA_{S} }} + \frac{{M_{1}^{2} }}{{2EJ_{1} }}} \right)} dx \hfill \\ \int_{0}^{{\frac{\pi }{2}}} {\left( {\frac{{M_{C} \left( \theta \right)^{2} }}{{2EJ_{2} }} + \frac{{N_{C} \left( \theta \right)^{2} }}{{2EA_{S} }} + \frac{{Q_{C} \left( \theta \right)^{2} }}{{2GA_{S} }}} \right)} Rd\theta \hfill \\ \end{gathered}$$

where, $E$ is the elastic modulus and the shear modulus $G = {E \mathord{\left/ {\vphantom {E {\left( {2\left( {1 + \mu } \right)} \right)}}} \right. \kern-\nulldelimiterspace} {\left( {2\left( {1 + \mu } \right)} \right)}}$. $\mu$ is the Poisson’s ratio.$A_{S}$ is the cross-sectional area. $J_{1}$ and $J_{2}$ are the elastic section modulus of the straight and curved segments, respectively. They can be determined as

5 $$\begin{gathered} J_{2} = J_{1} + \alpha^{2} A_{S} \hfill \\ J_{2} = \frac{{\pi d^{4} }}{64} + \alpha^{2} A_{S} \hfill \\ \alpha = R - d^{2} \left( {8R\left( {1 - \sqrt {1 - \left( \frac{d}{2R} \right)^{2} } } \right)} \right)^{ - 1} \hfill \\ \end{gathered}$$

Since the geometry and axial load are assumed to be symmetric, the deformation condition at point A is given as

6 $$\theta_{A} = \frac{\partial U}{{\partial M_{1} \left( t \right)}} = 0$$

Substituting Eq. (4) into Eq. (6), the moment $M$ is obtained as

7 $$M_{1} = \frac{{\left( {\pi - 2} \right)R^{2} J_{1} F_{k} }}{{4J_{2} L + 2J_{1} \pi R}} = k_{0} F_{k}$$

where $k_{0} = {{\left( {\pi - 2} \right)J_{1} R^{2} } \mathord{\left/ {\vphantom {{\left( {\pi - 2} \right)J_{1} R^{2} } {\left( {2LJ_{2} + \pi RJ_{1} } \right)}}} \right. \kern-\nulldelimiterspace} {\left( {2LJ_{2} + \pi RJ_{1} } \right)}}$. Substituting Eq. (7) into Eq. (4), the total potential energy $U$ can be obtained as

8 $$U = \left( \begin{aligned} \frac{{2k_{0}^{2} L}}{{EJ_{1} }} + \frac{L}{{2EA_{S} }} + \frac{R\pi }{{8EA_{S} }} + \frac{R\pi }{{8GA_{S} }} \hfill \\ + \frac{R}{{2EJ_{2} }}\left( {\frac{\pi }{2}\left( {2k_{0} - R} \right)^{2} + 2R\left( {2k_{0} - R} \right) + \frac{{R^{2} \pi }}{4}} \right) \hfill \\ \end{aligned} \right)N\left( t \right)^{2}$$

Then, the linear elastic deformation $\Delta L$ of the round link can be expressed as

9 $$\Delta L = \frac{\partial U}{{\partial N}} = \Delta L_{1} + \Delta L_{2} + \Delta L{}_{3}$$

where $\Delta L_{1}$,$\Delta L_{2}$,and $\Delta L{}_{3}$ are defined as

10 $$\left\{ \begin{gathered} \Delta L_{1} = \left( {\frac{{2k_{0}^{2} L}}{{EJ{}_{1}}} + \frac{L}{{2EA_{S} }}} \right)F_{k} \hfill \\ \Delta L_{2} = \left( {\frac{R\pi }{{8EA_{S} }} + \frac{R\pi }{{8GA_{S} }}} \right)F_{k} \hfill \\ \Delta L{}_{3} = \frac{R}{{2EJ_{2} }}\left( {\frac{\pi }{2}\left( {2k_{0} - R} \right)^{2} + 2R\left( {2k_{0} - R} \right) + \frac{{R^{2} \pi }}{4}} \right)F_{k} \hfill \\ \end{gathered} \right.$$

The relationship between the linear elastic deformation and the equivalent elastic force is therefore established.

**Figure 4:**
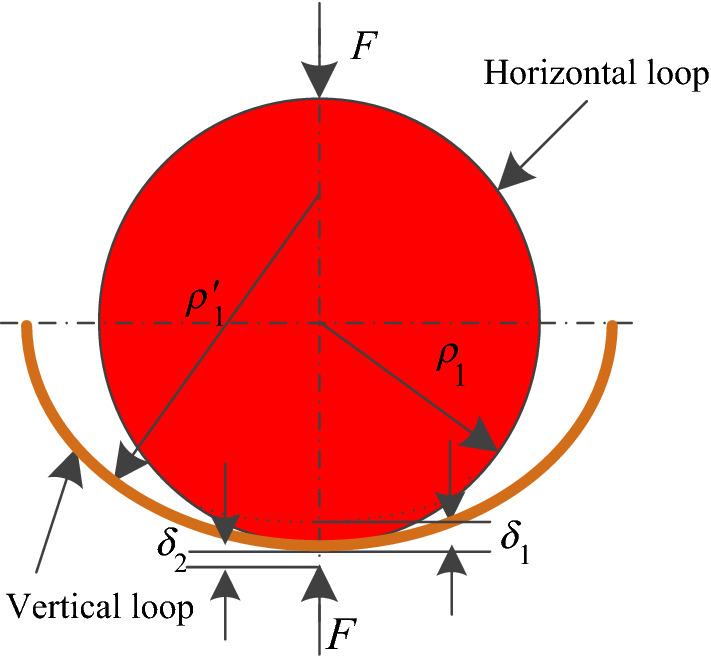
Diagram of local indentation deformations in the round link chain.

For the nonlinear elastic deformation due to contact, the Hertzian contact theory is used to derive the relationship between the indentation and the equivalent elastic force. As show in Fig. 4, the local indentation is induced between the adjacent round links subject to heavy axial loads. The principal radius of curvatures where the horizontal and vertical loops come into contact are defined as $\rho_{1}$ and $\rho_{1}^{\prime }$, respectively. The other principal radius of curvatures are defined as $\rho_{2}$ and $\rho_{2}^{\prime }$, respectively.

As the vertical and horizontal loops have the same geometry, the sum of the curvatures $\rho$ follows that^12^:

11 $$\left\{ \begin{gathered} \rho = \frac{1}{2}\left( {\frac{1}{{\rho_{1} }} + \frac{1}{{\rho_{2} }} + \frac{1}{{\rho_{1}^{\prime } }} + \frac{1}{{\rho_{2}^{\prime } }}} \right) = \frac{4R}{{d\left( {2R + d} \right)}} \hfill \\ \rho_{1} = \rho_{2} = \frac{d}{2} \hfill \\ \rho_{1}^{\prime } = \rho_{2}^{\prime } = - \left( {R + \frac{d}{2}} \right) \hfill \\ \end{gathered} \right.$$

The local indentation is assumed as circles, and the radius $e$ is much smaller than that of the curvature at the contact point. Hence, according to Hertzian contact theory, the radius $e$ and the max stress in the contact area $\sigma_{\max }$ can be given as

12 $$\left\{ \begin{gathered} e = \left( {\frac{{3\pi F_{k} \left( {k_{1} + k_{2} } \right)}}{4\rho }} \right)^{\frac{1}{3}} \hfill \\ \sigma_{\max } = \frac{{3F_{k} }}{{2\pi e^{2} }} \hfill \\ \end{gathered} \right.$$

where the parameter $k_{1} = k_{2} = {{\left( {1 - \mu^{2} } \right)} \mathord{\left/ {\vphantom {{\left( {1 - \mu^{2} } \right)} {\left( {\pi E} \right)}}} \right. \kern-\nulldelimiterspace} {\left( {\pi E} \right)}}$. The stress in the area of contact is given as follows:

13 $$\sigma_{{\left( {x,y} \right)}} = \sigma_{\max } \sqrt {1 - \frac{{x^{2} }}{{e^{2} }} - \frac{{y^{2} }}{{e^{2} }}}$$

The local deformations $\delta_{1}$ and $\delta_{2}$ at the contact area between the horizontal and vertical loops, respectively, are equal on the basis of symmetry. According to the geometrical relationship, the total contact-induced indentation deformation $\delta$ is obtained as

14 $$\delta = \delta_{1} + \delta_{2} = \left( {k_{1} + k_{2} } \right)\iint {\frac{{\sigma_{{\left( {x,y} \right)}} }}{r}ds} = \sqrt[3]{{\frac{{9RF_{k}^{2} \left( {1 - \mu^{2} } \right)^{2} }}{{E^{2} d\left( {2R + d} \right)}}}}$$

Assuming the displacement at point A of the round link is $x_{A}$, which is a combination of the linear elastic deformation and the contacting indentation, the total displacement of the round link chain with n links $x$ can be derived as

15 $$x = nx_{A} = 2n\left( {\Delta L_{1} + \Delta L_{2} + \Delta L_{3} } \right) + \left( {n - 1} \right)\delta$$

The equivalent nonlinear elastic force $F_{k}$ can then be expressed as a function of the displacement $x$. The equivalent nonlinear stiffness can also be obtained as

16 $$k_{e} = \left[ {\frac{\partial x}{{\partial F_{k} }}} \right]^{ - 1}$$

### Experimental identification of the nonlinear damping

According to Eq. (1), the equivalent nonlinear damping for the round link chain can be expressed as

17 $$c_{e} = \frac{{F - F_{m} - F_{k} }}{{\dot{x}}}$$

Since the inertial force $F_{m}$ and equivalent nonlinear elastic force $F_{k}$ have been expanded with respect to the displacement $x$, the equivalent nonlinear damping $c_{e}$ can be formulated, once the external force $F$ and displacement $x$ are tested in the experiment. This paper is focused on the round link chain in the scraper conveyer. In the practical working state, the fluctuating excitation is primarily induced by rotation of the driving system at some speed and initial deformations of the chain can also be observed due to transported coals. Thus, the external excitation can be taken as a simple harmonic motion, defined as

18 $$x = x_{0} + A\sin \left( {\omega t} \right)$$

where $A$ is the amplitude of the fluctuating displacement, $\omega$ is the angular velocity, and $x_{0}$ is the initial deformation due to the pretension force. Thus, the output force corresponding to the excitation can be defined as

19 $$F = F_{0} + F_{A} \sin \left( {\omega t + \varphi } \right)$$

where $F_{0}$ is the pretension force. $F_{A}$ is the amplitude of the pulsatile force response, and $\varphi$ is the phase variation between the force and the displacement.

The nonlinear dissipated force $F_{c}$ and corresponding equivalent nonlinear damping $c_{e}$ can be obtained as

20 $$\left\{ \begin{gathered} F_{e} = F_{0} + F_{A} \sin \left( {\omega t + \varphi } \right) + m\omega^{2} A\sin \left( {\omega t} \right) - F_{k} \hfill \\ c_{e} = \frac{{F_{c} }}{{\omega A\cos \left( {\omega t} \right)}} \hfill \\ \end{gathered} \right.$$

It can be found from Eq. (20) the equivalent nonlinear damping is a function of exciting frequency $\omega$ and the amplitude of the fluctuating displacement $A$. In this paper, the polynomial approximation method is utilized to approximate the equivalent nonlinear damping $c_{e}$. Since the highest power of both $\omega$ and $A$ taken in $c_{e}$ is 2, as shown in Eq. (20), $c_{e}$ is expanded as

21 $$c_{e} = \alpha_{1} \omega^{2} + \alpha_{2} \omega + \alpha_{3} A^{2} + \alpha_{4} A + \alpha_{5}$$

where the coefficients $\alpha_{1}$ and $\alpha_{2}$ are connected to the square of the velocity. $\alpha_{3}$ and $\alpha_{4}$ are connected to the velocity. $\alpha_{5}$ corresponds to the energy dissipation due to the pretension force. With five or more different experimental cases, all the coefficients can be determined and the equivalent nonlinear damping is then obtained. In order to improve the accuracy of the identified results, more than five experimental cases are used and the least square method for multiple variables are employed. Assuming there are *M*(M > 5) pairs of efficient test data and the fitted damping is $\overline{c}_{e}$, then the least square sum of the deviations $\varphi$ can be expressed as

22 $$\begin{aligned} \varphi & = \sum\limits_{i = 1}^{M} {\left( {c_{e} - \overline{c}_{e} } \right)^{2} } \\ & = \sum\limits_{i = 1}^{M} {\left( {c_{ei} - \alpha_{1} x_{1i} - \alpha_{2} x_{2i} - \alpha_{3} x_{3i} - \alpha_{4} x_{4i} - \alpha_{5} } \right)^{2} } \\ \end{aligned}$$

where $x_{1i}$,$x_{2i}$,$x_{3i}$ and $x_{4i}$ are $\omega_{i}^{2}$,$\omega_{i}$,$A_{i}^{2}$ and $A_{i}$, respectively. To ensure $\varphi$ is the minimal, the partial derivatives of $\varphi$ with respect to the unknown coefficients are taken to be zero. The corresponding system of linear equations in a simple form is obtained as

23 $$\bf GA = C$$

where the elements in the matrix $G$, and $C$ are defined as

24 $$\left\{ \begin{gathered} g_{kq} = \sum\limits_{i = 1}^{M} {x_{ki} x_{qi} } \hfill \\ c_{q} = \sum\limits_{i = 1}^{M} {c_{ei} x_{qi} } \hfill \\ \end{gathered} \right.$$

where $x_{5i} = 1$. The coefficients for the nonlinear damping are then calculated.

## Experimental setup and scheme

**Figure 5:**
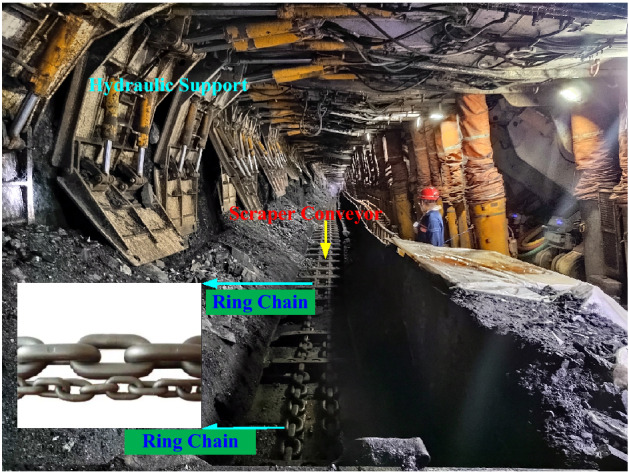
Actual use of underground ring chain in coal mine.

**Figure 6:**
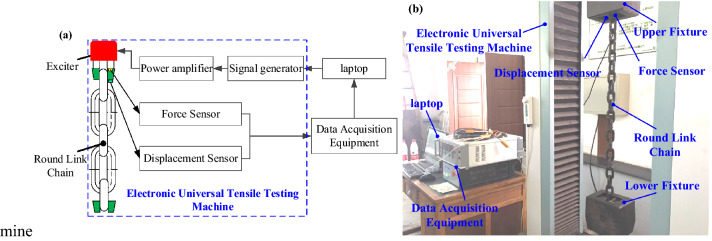
The schematic diagram of the test system.

In order to determine the coefficients for the equivalent nonlinear damping, an electronic universal tensile testing machine was employed to perform the dynamic tensile tests on the 14 × 50 round link chain used in mining applications. The circular chain is extracted from the coal mine of Shandong Energy Group. Figure 5 is the actual working condition of the circular chain used in the coal mine scraper conveyor. The schematic diagram of the experiment system is illustrated in Fig. 6. Harmonic displacement signals are generated by a signal generator controlled by a laptop. The signal is magnified by a power amplifier and drives the exciter to stretch the round link chain. In order to simulate practical loading conditions, a preload is firstly applied to ensure the round link chain is tensile condition in the test. The initial displacement, amplitude and frequency of the fluctuating load are set in the laptop. The total tensile force and displacement are measured by force sensor and displacement sensor, respectively. The measured signals are post processed by the data acquisition equipment and analyzed in the laptop.

**Figure 7:**
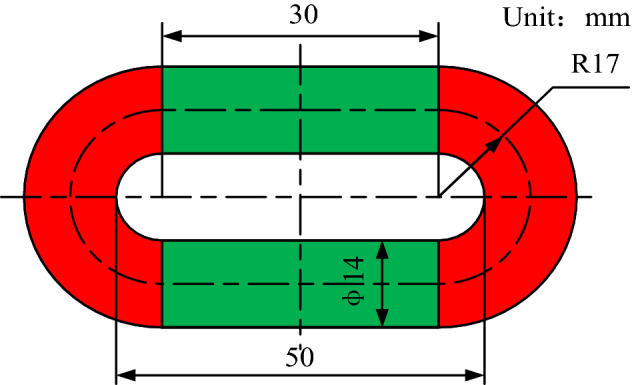
Dimensions of the round link.

The round link chain in the test is made of 25MnSi steel (*E* = 210 Gpa) and the mass per unit length is *m* = 4.0 kg/m. The geometrical dimensions of the round link chain used in the tests are shown in Fig. 7. The number of the round links between the fixtures is 16 (*n* = 16), and the effective length of the chain between the fixtures was 800 mm. The ambient temperature during the test is 23 °C.

## Results and discussion

### The equivalent nonlinear stiffness

**Figure 8:**
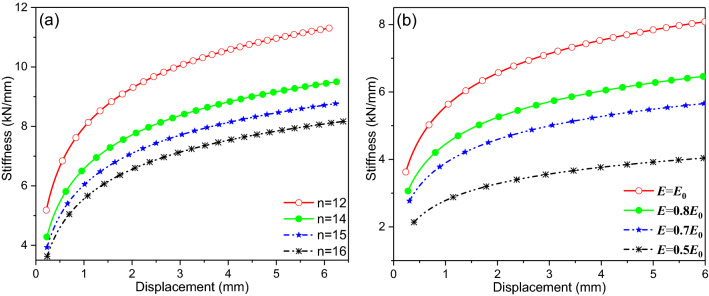
Parameter effects on the nonlinear stiffness, (**a**) effects of the total length and (**b**) effects of the elastic module with *E*_0_ = 2.1 × 10^5^ Mpa and *n* = 16.

In this part, the proposed theoretical model is employed to formulate the nonlinear stiffness of the 14 × 50 round link chain in the test. As shown in Fig. 8a, the stiffness of the round link chain is nonlinear with respect to the displacement *x*, especially when *x* is in the range from 0 to 3 mm. That is, when the axial deformation of each link is 0–0.3 mm, the nonlinearity of the stiffness is the strongest. With increase in the length of the chain, the stiffness and the nonlinearity are both weakened. When the displacement is large enough, the stiffness of the chain is inversely proportional to the length, which is the case for the linear elastic problems.

The influences of the material parameters on the stiffness of the chain are shown in Fig. 8b. A rise in the elastic module seems to reinforce the stiffness of the chain. Nevertheless, the stiffness is not any more proportional to the elastic module. The stiffness tends to be linear, when the material is more elastic.

### The equivalent nonlinear damping

In order to obtain the coefficients for the equivalent nonlinear damping, a series of experiments are performed, following the setup described in part 3. One of the tests is presented in detail to demonstrate the detailed identification procedure for the coefficients. In the case, $x_{0}$, $A_{0}$, $\omega$ and $k_{0}$ are taken as 2.2 mm, 0.25 mm, 1.884 rad/s and 0.08973 mm/s, respectively. Thus, the displacement excitation is expressed as

25 $$x = \left\{ \begin{gathered} 0.08973t{\kern 1pt} {\kern 1pt} {\kern 1pt} {\kern 1pt} {\kern 1pt} {\kern 1pt} {\kern 1pt} {\kern 1pt} {\kern 1pt} {\kern 1pt} {\kern 1pt} {\kern 1pt} {\kern 1pt} {\kern 1pt} {\kern 1pt} {\kern 1pt} {\kern 1pt} {\kern 1pt} {\kern 1pt} {\kern 1pt} {\kern 1pt} {\kern 1pt} {\kern 1pt} {\kern 1pt} {\kern 1pt} {\kern 1pt} {\kern 1pt} {\kern 1pt} {\kern 1pt} {\kern 1pt} {\kern 1pt} {\kern 1pt} {\kern 1pt} {\kern 1pt} {\kern 1pt} {\kern 1pt} {\kern 1pt} {\kern 1pt} {\kern 1pt} {\kern 1pt} {\kern 1pt} {\kern 1pt} {\kern 1pt} {\kern 1pt} {\kern 1pt} {\kern 1pt} {\kern 1pt} {\kern 1pt} {\kern 1pt} {\kern 1pt} {\kern 1pt} {\kern 1pt} {\kern 1pt} {\kern 1pt} {\kern 1pt} {\kern 1pt} {\kern 1pt} {\kern 1pt} {\kern 1pt} {\kern 1pt} {\kern 1pt} {\kern 1pt} {\kern 1pt} {\kern 1pt} {\kern 1pt} {\kern 1pt} \left( {0 \le t \le t_{0} } \right) \hfill \\ 2.2 + 0.25\sin \left( {1.884t} \right){\kern 1pt} {\kern 1pt} {\kern 1pt} {\kern 1pt} {\kern 1pt} {\kern 1pt} {\kern 1pt} \left( {t_{0} < t} \right) \hfill \\ \end{gathered} \right.{\kern 1pt} {\kern 1pt} {\kern 1pt}$$

**Figure 9:**
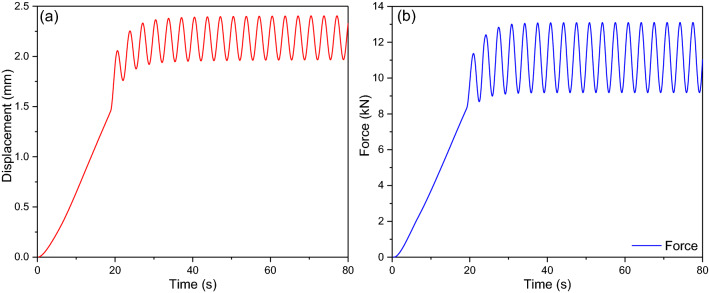
The loading and responses in time domain.

Figure 9a shows time-varying curves for the total displacement of the round link chain, and the dynamic force responses are measured with the force senor, as shown in Fig. 9b. With increment in the deformation of the round link chain, the nonlinearity is weakened. Overall, the frequency of the force responses keeps in agreement with that of the exciting displacement. This phenomenon and the variation of the stiffness to the displacement coincide mutually.

**Figure 10:**
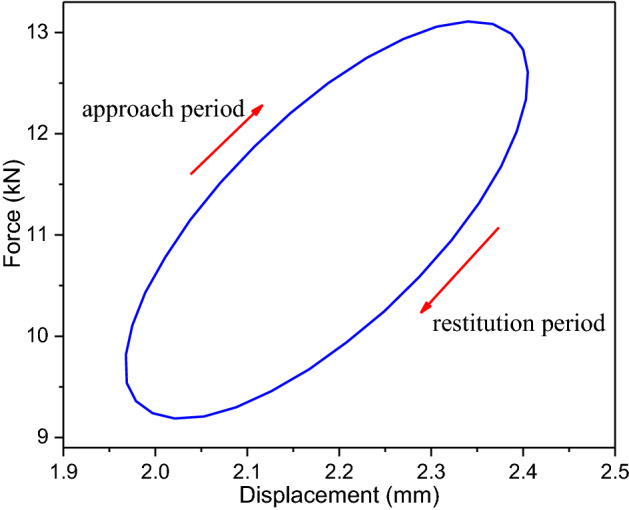
Force–displacement curve.

The relationship between the force responses and the displacement excitation in the steady state is obtained, as shown in Fig. 10. The approach track is distinct from the restitution one, which implies that significate energy dissipation is observed in the vibration of the round link chain. Therefore, it is necessary to involve the damping property into the dynamic model of the round chain under axial excitation, especially if the load is relatively large, like the loads in the mining engineering.

According to the measured data for the dynamic force response, a fitting function in waveform is employed to derive the formula of the force with respect to time *t*, expressed as

26 $$\begin{aligned} F & = F_{0} + F_{A} \sin \left( {\omega t + \phi } \right) \\ & = 11117.21 + 19522.1\;\sin \left( {1.883t - 0.8350} \right) \\ \end{aligned}$$

It is worth noting that R-square for the fitting is equal to 0.9999 and the fitting is therefore accurate enough. Now that the displacement, the geometrical and material parameters for the tested round link chain are determined, then the equivalent elastic force of the chain can also yield based on Eq. (15). One can calculate the elastic force and then submit it to Eq. (20) at each time step to determine the damping. Alternatively, to simplify the calculation, the formulation of the elastic force can also be fitted in the similar way. According to Eq. (20), the dissipated force and corresponding damping coefficient can be derived by using the property of the trigonometric functions. In this case, $F_{c}$ and $c_{e}$ can be expressed as

27 $$\left\{ \begin{gathered} F_{c} = F_{\alpha c} \cos \left( {\omega t} \right) = 1335\cos \left( {1.884t} \right) \hfill \\ c_{e} = \frac{{F_{C} }}{{\omega A\cos \left( {\omega t} \right)}} = {{2834.4\;{\text{N}}\;{\text{s}}} \mathord{\left/ {\vphantom {{2834.4\;{\text{N}}\;{\text{s}}} {{\text{mm}}}}} \right. \kern-\nulldelimiterspace} {{\text{mm}}}} \hfill \\ \end{gathered} \right.$$

As expressed in Eq. (21), the nonlinear damping depends on the amplitude and frequency of the external excitation source. A series of experiments are needed to get the coefficients in Eq. (21). Hence, with the same experimental model (the 14 × 50 round link chain), 9 different tests were implemented based on the aforementioned experimental setup. Corresponding damping properties are determined with the similar framework presented ahead, as listed in Table 1.

**Table 1:** Damping of the chain under different loading frequencies.

Test number	$\omega$/rad/s	$X_{0}$/mm	$A$/mm	$F_{ac}$/N	$c_{e}$/(N s/mm)
1	1	2.2	0.25	8000	5095.5
2	4	2.2	0.25	1100	3503.2
3	6	2.2	0.25	1335	2834.4
4	8	2.2	0.25	1465	2332.8
5	10	2.2	0.25	1542	1964.3
6	6	2.15	0.2	985	2614.1
7	6	2.25	0.3	1643	2906.9
8	6	2.3	0.35	2017	3058.8
9	6	2.35	0.4	2329	3090.5

A two-variable second-order polynomial equation is defined to fit the equivalent damping of the round link chain, as shown in Eq. (21). According to Eq. (23), the data in Table. 1 is utilized to calculate the coefficients in Eq. (21). The coefficients are finally obtained as

28 $$\left[ {\alpha_{1} {\kern 1pt} {\kern 1pt} \alpha_{2} {\kern 1pt} {\kern 1pt} \alpha_{3} {\kern 1pt} {\kern 1pt} \alpha_{4} {\kern 1pt} {\kern 1pt} \alpha_{5} } \right] = \left[ {28.02{\kern 1pt} {\kern 1pt} {\kern 1pt} {\kern 1pt} {\kern 1pt} {\kern 1pt} {\kern 1pt} - 651.57{\kern 1pt} {\kern 1pt} {\kern 1pt} {\kern 1pt} {\kern 1pt} {\kern 1pt} {\kern 1pt} - 8386.50{\kern 1pt} {\kern 1pt} {\kern 1pt} {\kern 1pt} {\kern 1pt} {\kern 1pt} {\kern 1pt} {\kern 1pt} {\kern 1pt} 7394.84{\kern 1pt} {\kern 1pt} {\kern 1pt} {\kern 1pt} {\kern 1pt} {\kern 1pt} {\kern 1pt} {\kern 1pt} {\kern 1pt} 4378.89} \right]$$

**Figure 11:**
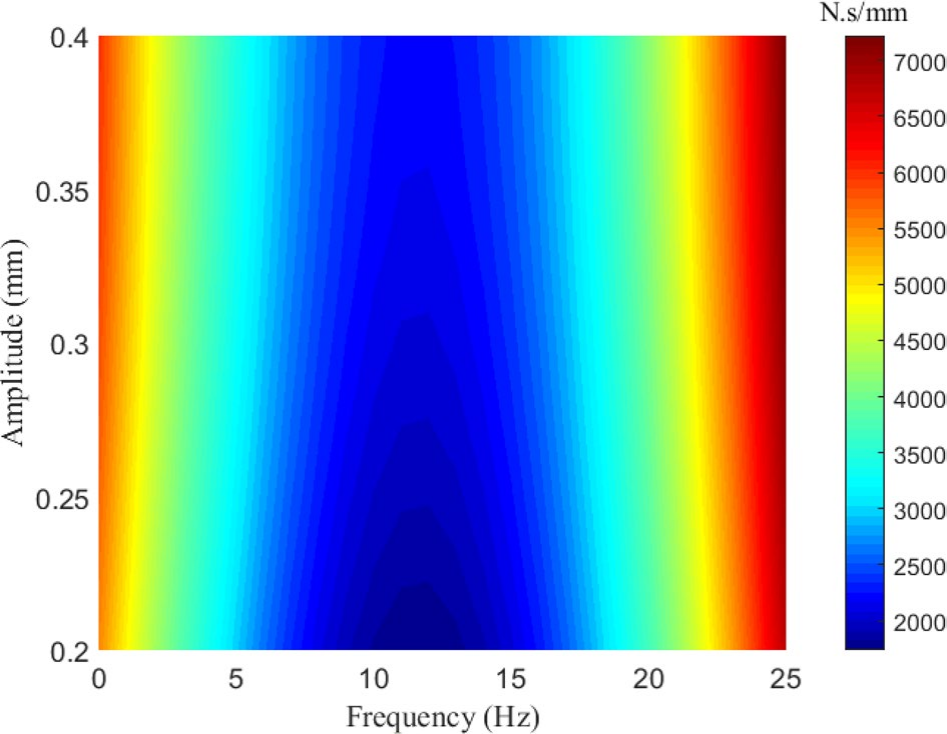
The nonlinear damping properties with respect to vibration amplitude and frequency.

As shown in Fig. 11, the damping is significant at low frequencies, while increase in frequency leads to decrease in the damping. However, when the frequency rises over about 12.5 Hz, the damping increases rapidly and it tends to be more significant at high frequencies. It may be because that the energy dissipation mainly lie in the plastic deformation of the round link in the low frequency range, while at high frequencies, friction and pounding play a major role in dissipating energy.

### Validation of the prediction model

**Figure 12:**
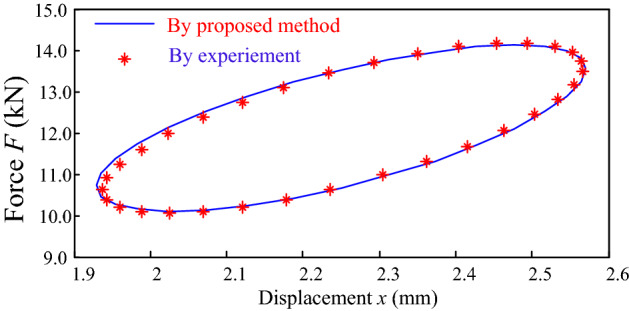
Comparison between the measured results and the prediction curve.

In order to validate the proposed framework for determination of the dynamic parameters of the round link chain, another test is implemented. Totally different key parameters are taken for the simulated harmonic displacement load to make sure the validation is accurate. The initial displacement $x_{0} = 2.25\;{\text{mm}}$, the loading frequency $f = 7\;{\text{Hz}}$, and the amplitude $A = 0.32\;{\text{mm}}$. Then displacement excitation turns to be $x = 2.25{ + 0}{\text{.32}}\sin \left( {14\pi t} \right)$. According to Eq. (15), the elastic force $F_{k}$ is obtained, and the damping can be given by submitting $A = 0.32\;{\text{mm}}$ and $f = 7\;{\text{Hz}}$ into Eq. (21). According to Eq. (1), the force response is then determined. The curves for the force response to the exciting displacement are employed to analyze the accuracy of the proposed method. The proposed result is compared with the experimentally measured curve, as shown in Fig. 12. Good agreement is observed between the proposed and experimental results.

## Conclusion

This paper presents a new framework to identify the nonlinear stiffness and damping properties of a round link chain under axial dynamic excitation. A modified nonlinear viscoelastic model is developed to study vibrations of the chain with numbers of round links. Based on linear elastic theory and contacting models, a theoretical model is established for the equivalent nonlinear stiffness, including elastic and contacting deformation effects. A series of dynamic experiments are performed and accordingly, the least square fitting method is employed to determine the equivalent damping of the chain. Another test case with totally different loading parameters is designed to validate the proposed framework and the proposed results agree well with the experimental ones. The effects of key material and loading parameters on the nonlinear stiffness and damping are examined. It is found with small dynamic deformations the stiffness is strong nonlinear to the deformation, and the nonlinearity will turn to small if the deformation or the length of the chain increase, or the elasticity of the chain decrease. Due to transformation of the main manners of the energy dissipation, the nonlinear damping is firstly decreased with rise in the loading frequency. However, when the frequency increase over a critical one, the damping turns to increase. That is, the nonlinear damping is significant at both low and high frequencies, while it is relatively small in the middle frequency range. The developed identification framework and proposed results can provide some physical insights into the dynamic properties of the round chain and can help improve the performance of the machines such as the scraper conveyor.

## Data Availability

Due to the ongoing project of the team, the data sets obtained during the current study are temporarily not public, but can be obtained from the corresponding authors according to reasonable requirements.
